# Development of an Electrochemical Sensor for SARS-CoV-2 Detection Based on Loop-Mediated Isothermal Amplification

**DOI:** 10.3390/bios13100924

**Published:** 2023-10-11

**Authors:** Ane Rivas-Macho, Unai Eletxigerra, Ruth Diez-Ahedo, Ángela Barros, Santos Merino, Felipe Goñi-de-Cerio, Garbiñe Olabarria

**Affiliations:** 1 Gaiker, GAIKER Technology Centre, Basque Research and Technology Alliance, 48170 Zamudio, Spain; 2 Molecular Biology and Biomedicine PhD Program, University of the Basque Country UPV/EHU, 48940 Leioa, Spain; 3 Surface Chemistry and Nanotechnologies Unit, Tekniker, 20600 Eibar, Spain; 4 Electricity and Electronics Department, University of the Basque Country UPV/EHU, 48940 Leioa, Spain

**Keywords:** loop-mediated isothermal amplification (LAMP), electrochemical detection, SARS-CoV-2, biosensor

## Abstract

The pandemic caused by the severe acute respiratory syndrome coronavirus 2 (SARS-CoV-2) caused more than 6 million deaths all over the world, demonstrating the need for a simple, fast and cost-effective point-of-care (POC) test for the detection of the virus. In this work, we developed an electrochemical sensor for SARS-CoV-2 virus detection on clinical samples based on loop-mediated isothermal amplification (LAMP). With the development of this novel sensor, the time of each measurement is significantly reduced by avoiding the DNA extraction step and replacing it with inactivation of the sample by heating it at 95 °C for 10 min. To make the reaction compatible with the sample pre-treatment, an RNase inhibitor was added directly to the premix. The LAMP product was measured in a novel, easy-to-use manufactured sensor containing a custom-made screen-printed carbon electrode. Electrochemical detection was performed with a portable potentiostat, and methylene blue was used as the redox-transducing molecule. The developed sensor achieved a limit of detection of 62 viral copies and was 100% specific for the detection of the SARS-CoV-2 virus. The performance of the electrochemical sensor was validated with nasopharyngeal samples, obtaining a sensibility and specificity of 100% compared to the gold standard RT-PCR method.

## 1. Introduction

The global outbreak caused by the severe acute respiratory syndrome coronavirus 2 (SARS-CoV-2) was declared a pandemic by the World Health Organization (WHO) in March 2020. From December 2019 to April 2023, this virus caused 6.9 million deaths due to the Coronavirus Disease (COVID-19) Pandemic [1]. The availability of antigen-based lateral flow immunochromatography tests for mass population screening in conjunction with vaccination meaningfully reduced the mortality rate. However, the sensitivity of antigen-based tests is significantly lower than RT-PCR-based methods [2]. Therefore, rapid, accurate and low-cost diagnosis of SARS-CoV-2 remains highly important in diverse populations, such as those carried out in nursing homes, self-testing or in hospitals in countries with limited laboratory facilities. The standard method used for the detection of SARS-CoV-2 is the RT-PCR method [3]. This is the most used method for diagnostics of clinical pathogens due to its high specificity and sensitivity. However, for a point-of-care (POC) application, the PCR is hardly scalable due to the sample extraction process prior to amplification [2] and the cycling temperatures [4]. As an alternative to PCR, several isothermal amplification methods have been developed; one of the most reported is loop-mediated isothermal amplification (LAMP).

The LAMP technique allows DNA amplification at a single temperature [5], and the results can be detected by fluorescence [6] or turbidimetry [7]. These methods require complex manual procedures and laboratory equipment, hindering the integration of the reading in a portable device. Most recent publications on the POC-based LAMP technique for SARS-Cov-2 have focused on colourimetric detection because it is a rapid and simple diagnostic option [8,9,10,11,12,13]. The authors used a pH-dependent commercial master mix based on a visible pH indicator that triggers a colour change in the reaction from pink to yellow when the target RNA is amplified. However, all of them were performed with RNA extracted and purified from clinical samples with different commercial kits.

In pandemic situations, RNA extraction is a major bottleneck and challenge to overcome because sample treatment in this process is a crucial step for LAMP sensor performance in a point-of-care scenario. Indeed, the sample treatment must be simplified to be performed on-site. For example, extraction methods based on a silica column are incompatible with POC testing due to the necessity of laboratory equipment, such as a bench-top centrifuge [14]. Another simpler alternative is magnetic-bead-based methods, but the extraction yield is less efficient due to several beads washing steps that are still time-consuming [8,9].

In the last year, extraction-free sample treatments have arisen as the best option for POC testing. These treatments require only a simple heating step for the extraction of viral RNA from the clinical sample [15] or the addition of an extra reagent in the amplification mix for the neutralisation of the inhibitors present in the sample [16]. In this regard, most of the reported results are performed with commercial RNA and artificially spiked samples [17]. According to our results, these samples behave differently from naturally contaminated samples containing RNases that can be activated during the LAMP reaction, decreasing the amount of target RNA and giving false negative results. Another major disadvantage of these extraction-free methods is that they are incompatible with the pH-sensitive colourimetric LAMP reaction due to the variety of pH found in clinical samples that can change the reaction colour even before the start of amplification, giving false positive results [18].

A good chance to overcome this drawback is to use alternative pH-independent detection systems. In this regard, electrochemical detection of LAMP products has been widely used in terms of POC testing [19,20]. These types of detection options have proven to have advantages, such as portability, low cost, robustness and feasibility for integration. [21]. Nevertheless, the electrochemical sensors reported for SARS-CoV-2 detection need a labelled receptor immobilised on the working electrode [22,23], which limits the achievement of a reliable, affordable and versatile platform for in situ detection. A recent review [24] includes those based not only on the detection of viral nucleic acid but also on immunoglobin, antigen and the entire viral particles, relying in all cases on the working electrode functionalisation. A receptor-free electrochemical transduction mechanism for SARS-CoV-2 detection in wastewater samples has been reported by R.G. Ramírez et al. [21] with the disadvantage that RT-LAMP is performed in two steps: one for cDNA generation and the other for cDNA amplification. In addition, the sensor has not been tested with viral RNA, entire viruses, spiked water or wastewater. Furthermore, the measurements have been performed with an unsealed sensor, unable to avoid cross-contamination effects, which are very common in LAMP-amplified products.

To solve all these drawbacks, we developed a novel electrochemical sensor encapsulated in a microfluidic chamber and compatible with an extraction-free method that has been validated with nasopharyngeal (NP) samples. Using a closed chamber guarantees complete wetting and an equal amount of sample over the printed electrodes, avoiding liquid evaporation. This facilitates the robustness of measurements and prevents external contamination. The detection is achieved by adding the redox-active Methylene-Blue (MB) molecule to the LAMP reaction, which intercalates into the double-stranded DNA [20]. When the MB is free in solution, the molecule diffuses onto the surface of the working electrode, and the redox reaction generates a current peak at specific potentials, indicative of a negative result. In the case of positive samples, in which the SARS-CoV-2 RNA is present, the MB molecule intercalates into the polymerised double-strand DNA. This limits the amount of free MB in the sample and restricts the electron transfer reaction by minimizing the intensity of the current peak [25]. 

The novelty of our developed sensor relies on the combination of an extraction-free clinical sample treatment with an electrochemical LAMP (EC-LAMP) performed inside a microfluidic chamber containing the electrodes for electrical signal monitoring. We have developed a new and optimised LAMP method using MB as a redox molecule and an RNase inhibitor, compatible with electrochemical measurement, which neutralises the activity of RNases present in extraction-free NP samples. 

The electrochemical detection was studied in an open configuration, without a chamber, for the optimisation of the reaction mix and closed, where the sample was injected into the microfluidic chamber. The electrochemical sensor can detect SARS-CoV-2 in clinical samples in less than 1 h, 10 min for sample treatment and 40 min for EC-LAMP. The novel manufactured sensor shows 100% specificity against other common respiratory viruses and a limit of detection of 62 viral copies. The performance of the electrochemical sensor was validated with NP samples, obtaining a sensibility and specificity of 100% compared to the gold standard RT-PCR method. In conclusion, the disposable microfluidic chamber integrating the sensor can be used for on-site detection of SARS-CoV-2 in NP samples, requiring only an inexpensive and portable heater and a miniaturized potentiostat. It provides an affordable and versatile platform for an easy-to-develop qualitative sensor in resource-limited or field settings.

## 2. Materials and Methods

### 2.1. Materials and Reagents

Extraction of the nucleic acids was performed with the QIAamp Viral RNA Mini Kit from Qiagen, Germany. RT-LAMP and RT-PCR primers were synthesized by Biomers, Germany. For the RT-qPCR, One Step PrimeScript III RT-qPCR Mix (Takara, Japan) was used. LAMP mixture buffer for isothermal amplification ThermoPol buffer (10X), MgSO_4_, dNTPs and Bst DNA polymerase large fragment were purchased from New England Biolabs, UK, and the Evagreen fluorescence dye was purchased from Biotium, EEUU and Maxima Reverse Transcriptase from Fisher Scientific, S.L., betaine, Methylene Blue (MB) and the Tris-EDTA buffer for the collection of the samples were acquired from Sigma Aldrich, UK. The RiboGuard RNase inhibitor was purchased from Lucigen, UK. Fluorescence emission was monitored with the CFX Connect Real-Time System (Bio-Rad, EEUU). The LAMP optimisation and LoD study were performed with quantified pure Amplirun SARS-CoV-2 RNA (Vircell). For the specificity assays, quantified pure RNA from several different relative viruses—Coronavirus OC43, Enterovirus 68, Rhinovirus, MERS coronavirus, Coronavirus SARS-2003 and a panel of 10 respiratory viruses (Amplirun Total Respiratory Viral Panel Control)—were acquired from Vircell, Spain. 

For the sensor cartridge fabrication, pressure-sensitive adhesive thin film ARseal^TM^ 90880 (Adhesive research), polycarbonate Lexan 8010 MC (Konig), Stand-alone female mini luer 10000701 (ChipShop), Male mini luer plug 10000030 (ChipShop), and double side adhesive tape 3M^TM^ 9088-200 were used.

### 2.2. Methods

#### 2.2.1. Clinical Samples and Spiked Samples

For the limit of detection assays, a commercial quantified synthetic SARS-CoV-2 RNA was used (Amplirun SARS-CoV-2 RNA Control). The negative NP samples were spiked with 1000 copies of the commercial RNA of the virus, and four serial half-dilutions were performed until 62.5 copies were reached.

The 26 NPs used in this work were from volunteers of the Gaiker Technology Centre employees. The samples were self-collected in 3 mL of 10 mM Tris-HCl, 1 mM EDTA, pH 8.0 (TE) buffer and stored at −80 °C until further use. Eleven of them were positive samples and 15 negative ones, and the results were confirmed and monitored by the RT-PCR method.

#### 2.2.2. RT-PCR Reaction

As a reference method, the RT-qPCR was used. The NP sample results were confirmed with this method. The extraction of the sample was performed with the QIAamp Viral RNA Mini Kit following the manufacturer’s instructions. The RT-qPCR reaction contains 10 µL of the One Step Prime Script III RT-qPCR Mix, 0.11µM of primers Forward (5′-GAC CCC AAA ATC AGC GAA AT-3′) and Reverse (5′-TCT GGT TAC TGC CAG TTG AAT CTG-3′) targeting the N gene recommended by the WHO [26], 0.2 µM of probe (5′-FAM-ACC CCG CAT TAC GTT TGG TGG ACC-BHQ1-3′), RNase/DNase free water and 5 µL of sample in a total reaction volume of 20 µL. The reaction was performed in a CFX Connect thermocycler as follows: 52 °C for 5 min, 95 °C for 10 s and 40 cycles of 95 °C for 5 s and 55 °C for 30 s acquiring in the green filter.

#### 2.2.3. Design of Primer Sets and Experimental Optimization

For the design of the SARS-CoV-2 specific LAMP primers, an initial in silico study was performed, completing alignments of the SARS-CoV-2 genome and related coronavirus with the BioEdit v7.0.5.3 software. When the target region was defined, the primers were designed with the software PrimerExplorerv5 and LAMP Designer. Twenty-two sets of primers were designed, two targeting the gene S, four against the ORF1ab region, six in the RdRp gene, three on the E gene and seven targeting the gene N.

For all the sets of primers, the RT-LAMP reaction was optimised testing temperature in a range of 60–65 °C, magnesium ion concentration between 4–8 mM and betaine concentration between 0–0.6 M. The optimized reaction for the selected ND3B primer set was performed in a final volume of 25 µL and consisted of 4µL of primers FIP and BIP (1.6 µM), LF and LB (0.8 µM) and F3 and B3 (0.2 µM), 2.5 µL of 10X Bst Large Buffer, 1.5 µL of MgSO_4_ 100 mM, 3.5 µL of dNTP solution mix, 2 µL of Betaine 5 M, 1.5µL of Bst Large 8 U µL^−1^, 0.2 µL of Evagreen, 0.1 µL of Maxima Reverse Transcriptase 200 U µL^−1^, RNase/DNase free water and 5 µL of sample. The amplification reaction was performed at 63 °C for 40 min and monitored in real-time with a CFX Connect thermocycler with 60-s intervals and reading with the green filter. For all RT-LAMP reactions, the time to detection (TTD) value was considered; this value represents the time at which fluorescence emission begins and amplification occurs.

Serial dilutions from 1000 to 16 copies of purified and quantified viral RNA were used for the Limit of Detection (LoD) assays. The different total RNA copies were tested in replicates of 10, and the LoD was established at the level where at least 95% of the replicates were positive. The specificity assay was tested with 1000 viral copies of related viruses and a panel of respiratory viruses listed in Table 1 as exclusive. As an inclusive strain, the SARS-CoV-2 genome was used. For the pool of respiratory virus, the extraction of the nucleic acids is necessary, and this extraction was performed with the QIAamp Viral RNA Mini Kit following the manufacturer’s instructions.

#### 2.2.4. Clinical Sample Preparation and RNA Extraction

For the development of an easy-to-use POC-compatible extraction protocol, two fast methods were tested. As the reference extraction protocol, the silica column-based method (QIAamp Viral RNA Mini Kit) was used following the manufacturer’s recommendations. The first fast extraction protocol was to heat the sample at 95 °C for 10 min using a heat-block. The second strategy was a free extraction method to test the sample directly for amplification without any pre-treatment. In both strategies, 1 U µL^−1^ RiboGuard RNase inhibitor was added in the RT-LAMP premix to avoid degradation of the virus RNA during amplification. For the three protocols, a comparative study was performed with NP samples collected in TE. 

#### 2.2.5. Electrochemical RT-LAMP Reaction and Measurements

For the electrochemical detection, the RT-LAMP reaction contains the same reagents as mentioned before, replacing the Evagreen dye with the MB molecule at 8 µg mL^−1^. Electrochemical detection works by measuring the oxidation reaction in the electroactive molecule due to the interaction with the LAMP product. MB was chosen due to its well-known electroactive behaviour and the fact that it does not inhibit DNA amplification [27]. All the electrochemical measurements were performed using a DRP-STAT400 potentiostat from DropSens. The custom-made Screen-Printed Carbon Electrodes (SPCEs) are a geometrical variation of the C110 model, consisting of a circular carbon Working Electrode (2 mm diameter, WE), a carbon Counter Electrode (CE) and a silver pseudo-Reference Electrode (RE) optimised for low sample volumes.

Three consecutive scans were performed by cyclic voltammetry at a 100 mV s^−1^ scan rate in a potential window from –0.5 to 0 V, and the height of the oxidation peak of the last one was compared. The potential window was chosen to avoid the redox reaction of electropolymerisated species or electropolymerisation of MB [28]. Two different measurement procedures were followed. On the one hand, open configuration determinations, called “off-chip”, were performed directly by placing a 25 µL sample drop covering the three electrodes. On the other hand, measurements inside the sensor in a closed configuration, called “on-chip”, were performed by injecting 25 µL of sample inside of the sensor chamber. In both cases, the voltammetry cycles were obtained immediately after the sample was in contact with the electrodes to avoid the interference of MB adsorption on the electrode surfaces [29].

## 3. Results

### 3.1. Optimisation of RT-LAMP Reaction

To develop a specific and sensible RT-LAMP reaction, 22 sets of LAMP primers were designed using two different software programs, PrimerExplorerv5 [30] and LAMP Designer [31]. The primers were designed to target four different regions of the SARS-CoV-2 genome corresponding to Nucleocapsid (N), Spike protein (S), Envelope protein (E) and the ORF1ab region. The RT-LAMP reaction was optimized for each set of primers attending the critical parameters of the reaction, testing different temperatures, magnesium ion concentration and betaine concentration. For the optimisation study, real-time monitoring of the reaction was performed, using Evagreen as the fluorescence intercalating dye. Of these 22 sets of primers, 5 were selected as the best candidates to continue with the optimization of the RT-LAMP reaction. This selection was performed considering a first screening of the LoD of each of the primer sets. 

According to the Foundation for Innovative and New Diagnostics (FIND), for verification of the LoD of SARS-CoV-2, serial dilutions from 1000 to 16 copies of quantified whole viral RNA were used to create a standardized dilution series including 10 replicates of eight dilutions. Table 2 shows the results of the LoD study for the five sets of primers previously selected. From the LAMP perspective, as from qPCR, the LoD can be defined as the lowest concentration of target analyte that can be detected with a defined level of confidence, with a 95% detection rate as the standard confidence level [32]. The best candidate was ND3B, with a LoD of 62 copies. The LoD of ND1-1, S.45, E.105 and ORF1ab.99 were 62, 125, 250 and 250 copies, respectively. 

**Figure 1:**
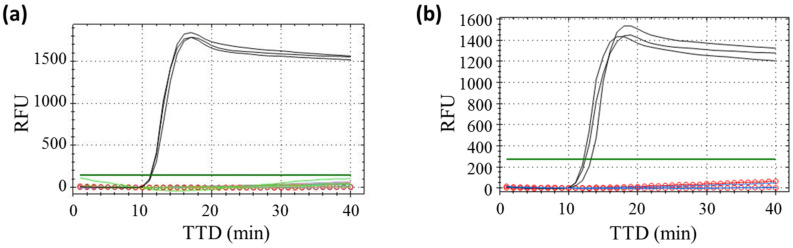
Specificity results for ND3B primer set. (**a**) Cross-reactivity assay against some related and common respiratory viruses. SARS-CoV-2 (Black), Coronavirus OC43 (Blue), Enterovirus 68 (Green), Rhinovirus (Purple), MERS coronavirus (Grey), Coronavirus SARS-2003 (Orange) and not template control (Red circles). (**b**) Specificity assay against a panel of other respiratory viruses (Blue). SARS-CoV-2 is represented in pink, and the non-template control is represented by red circles. All samples were run in triplicates. TTD is represented in minutes at the bottom, and RFU (Relative Fluorescence Units) is on the left.

The specificity of the reaction was tested against the viral RNA from other coronaviruses and related viruses, and a panel of respiratory viruses pooled and formulated in a viral transport medium (VTM), represented in Table 1. In Figure 1, it can be observed that the primer set ND3B was totally specific for the detection of the SARS-CoV-2 virus. That primer set was selected as the best candidate for the rest of the study.

### 3.2. Off-Chip Electrochemical Measurements

**Figure 2:**
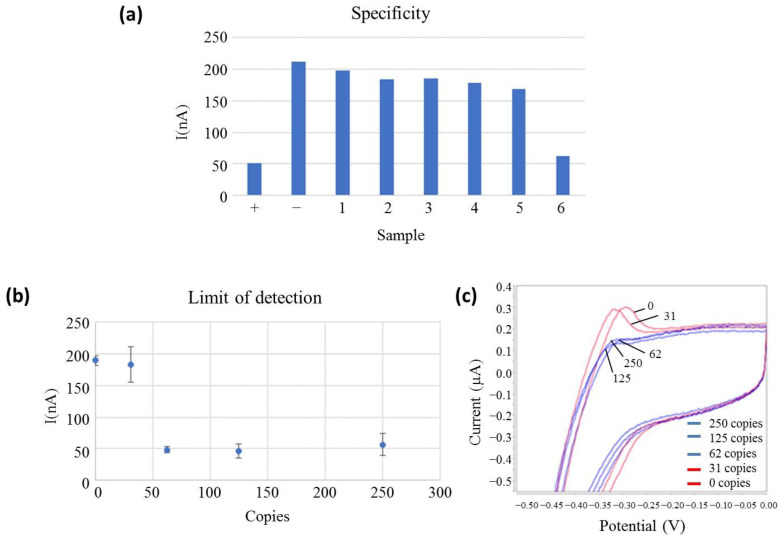
(**a**) Off-chip electrochemical measurements of the specificity assay. The bar height represents the measured oxidation peak current related to the MB oxidation reaction. The sample is represented at the bottom. SARS-CoV-2 (+), non-template control (−), Coronavirus OC43 (1), Enterovirus 68 (2), Rhinovirus (3), MERS coronavirus (4), SARS-2003 coronavirus (5) and Pool of respiratory viruses (6) and current peak (nA) are represented on the left side; (**b**) Electrochemical limit of detection measures. The copy number is represented at the bottom (Copies), and the current peak (nA) is represented on the left side. All samples are measured in triplicates, and the standard deviation is represented in bars; (**c**) voltammetry curves of the electrochemical limit of detection measures positive samples and negative samples in blue and red, respectively. Potential is represented at the bottom (V), and the current peak (mA) is represented on the left side.

The specificity and LoD of the off-chip EC-LAMP measurements were studied by placing a drop of the LAMP-amplified products over the electrode. As in Section 3.1, the specificity of the reaction was tested against RNAs from related coronaviruses or common respiratory viruses and a pool of respiratory viruses (Table 1). The off-chip electrochemical results can be observed in Figure 2a. The EC-LAMP was specific for the detection of SARS-CoV-2 RNA, obtaining results comparable to those obtained in the fluorescence assay (Figure 1), with a significantly lower signal for the positive sample (+) than for non-template control (-) and for the related exclusive viruses (samples 1–5). 

However, the respiratory virus pool (sample 6) presented the same low current signal as the positive (+) sample. The respiratory virus pool is a commercial product that contains supplemental components (other than the virus and VTM) that the manufacturer includes to preserve the stability of viruses during the lyophilisation process since this product is provided in a lyophilised format. As demonstrated in the Figure 1b, fluorescence-based LAMP is not interfered with this sample. Our hypothesis is that the false positive result obtained with the EC-LAMP was due to supplementary sample components that react with the MB, inhibiting its electroactivity. This fact was confirmed by several experiments in which the respiratory virus pool was directly mixed with MB, without any premix or amplification, and gave a positive signal. Next, all clinical samples tested were collected in TE buffer as VTM because it did not react with MB.

Apart from sample 6, the cathodic peak current of the non-template control was around 208 nA for the experimental conditions described, while a positive control was 50 nA and tested negative viruses provided current intensities in the range of 196–170 nA. Therefore, a current intensity ratio between 3 and 4 was obtained for positive and negative samples, showing a good specificity towards the target virus. In fact, considering for this specificity test, a cut-off value of the electrical current above 100 nA to be considered negative and the values obtained for negative samples in a one-sample t-test, a statistical t of 17.72 is obtained, which shows a confidence level higher than 99.5%. 

The LoD of the reaction was determined using serially diluted SARS-CoV-2 RNA and calculated as three times the value of the standard deviation (3SD) obtained in the absence of the target RNA. The reported LoD corresponded to the lower measured concentration with at least a 3SD difference with respect to the negative control. From Figure 2b,c, it is concluded that the developed test reached an analytical detection limit of 62 copies of viral RNA, the same result obtained with the fluorescence detection method. Figure 2b shows the error bars representing the standard deviation of each measurement for three replicates, clearly showing a >3SD gap between positive and negative samples. The signal of positive dilutions showed similar current intensities, even if the copy number of the target RNA decreased, as the electrochemical signal was read during the plateau phase at the end of the LAMP reaction. This is because after the whole reaction has concluded, the amplification products outweigh the influence of the initial DNA concentration. This result demonstrates that the LoD obtained with electrochemical detection is comparable to fluorescence detection.

### 3.3. Clinical Sample Pre-Treatment Assays

A crucial challenge for the development of a POC sensor is that the nucleic acid extraction method has to be simple, fast and efficient in order to be easily adaptable to the requirements of the sensor. In order to develop a simple extraction method, two strategies were carried out and compared to the silica column-based reference method. The first approach consisted of adding the sample directly into the amplification reaction without any treatment, and the second consisted of heating a volume of the NP sample at 95 °C for 10 min.

**Figure 3:**
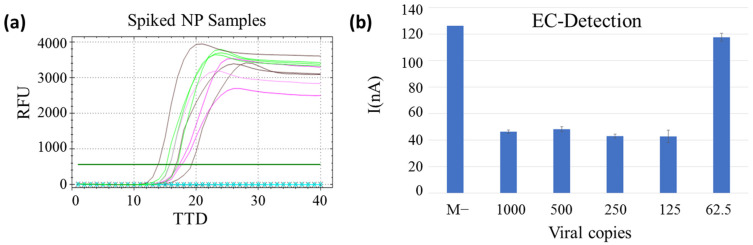
(**a**) Limit of detection of the extraction-free method with spiked samples. Positive controls (viral RNA) in pink, spiked NP samples in green (250 copies), brown (125 copies) and the blue circled line (62.5 copies) and the non-template control is represented by black crossed line. TTD is represented at the bottom, and RFU on the left. (**b**) Limit of detection of the extraction-free RT-LAMP by electrochemical detection. Copy number per reaction and negative clinical sample (M−) represented in the bottom and current peak (nA) on the left. All samples were measured in triplicates, and the standard deviation is represented in bars.

The limit of detection was studied for both approaches with NP-negative samples that were spiked with half-serially-diluted virus genome copy number. As represented in Figure 3a, the reaction was compatible with a free extraction method, and the limit of detection obtained for the non-treatment strategy was 125 copies for the fluorescence-based RT-LAMP. However, this LoD could not be tested for the second strategy (95 °C for 10 min) because the serially diluted RNA spiked into the NP samples was completely degraded during the heat treatment. 

The LoD study for the non-treatment method was also performed with the EC-LAMP to confirm compatibility with electrochemical measurements. As mentioned in Section 3.2, there is no difference in the intensity of the positive samples as this is an end-point measurement. These results were used to establish the limit of detection of the method at 125 viral copies for spiked clinical samples, as for fluorescent LAMP, demonstrating that the extraction-free strategy is compatible with electrochemical measurements. Differences in absolute figures of current intensities for spiked clinical samples and purified RNA, studied in Section 3.2, obey the nature of the sample, which provides different adsorption on the surface of the electrodes, responsible for the charge accumulation that varies both capacitive and electrical intensity estimation associated with the electroactivity of the MB.

To confirm the above results with non-spiked NP-positive samples, the two strategies were carried out and compared with the silica column-based reference extraction method. All the assays were performed with clinical NP samples that were positive for SARS-CoV-2 by RT-PCR. Three out of 11 NP-positive samples performed with the extraction-free method (not preheated) were not detected. As shown in Table 3, the heating treatment gave the same results as the reference method but improved the time required per sample to complete the process. These results can be explained by the fact that the heating process is able to release the RNA from the virus capsid, improving sensitivity and TTD. The limit of detection achieved with the extraction-free spiked NP samples was better than the already published for other electrochemical sensors [17]. The heat extraction method obtained better sensitivity results with real positive samples, suggesting that the clinical sensitivity obtained is similar to the reference extraction method. Considering all the results into account, the heat extraction method was the best option for the sample pre-treatment.

### 3.4. Sensor Design and Fabrication

An SCPE sensor was adapted with a low-cost disposable microfluidic chamber that was fabricated for measuring the EC-LAMP products. The chamber was easily fabricated by means of a sandwich configuration using two thin transparent biocompatible polymers: Polycarbonate (PC) and 142 µm thick medical grade double side pressure sensitive adhesive (PSA) film. A PSA film containing the cut-out designed chamber was pressure adhered over the SCPEs. To close the chamber, a PC film perforated with an inlet and an outlet was aligned and adhered over the PSA. The chamber design, inlet and outlet were designed and cut in a Silhouette Cameo^®^ precision cutting tool. Two connectors were aligned and glued with double-sided tape to the PC inlet/outlet. They allowed the integration of the fluidic chamber via pipette or tubing to a syringe or waste reservoir to control the sample inlet and outlet, respectively. Finally, the two plugs closed the inlet/outlet after pipetting the sample. 

**Figure 4:**
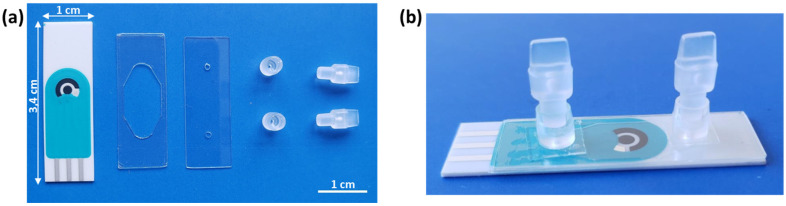
Pictures of (**a**) individual components of the measurement chamber. From left to right: SCPE, double-sided PSA film containing the cut-out chamber area, perforated PC with inlet/outlet, connectors and plugs. (**b**) Assembled measurement chamber over the SCPEs chip.

SCPEs were purchased and customised with a 2 mm WE in the middle of the ceramic substrate. The microfluidic chamber was designed in a proper size to avoid contact with the measurement electrodes and to allow the complete filling of the camera with 25 µL of sample. A closed configuration of the chamber was chosen to avoid cross-contamination between samples and to guarantee a complete wetting and an equal amount of sample over the electrode. A picture of all the components described for the assembly of the measurement chamber is shown in Figure 4a. Figure 4b shows the fully assembled chip.

### 3.5. On-Chip Measurements

**Figure 5:**
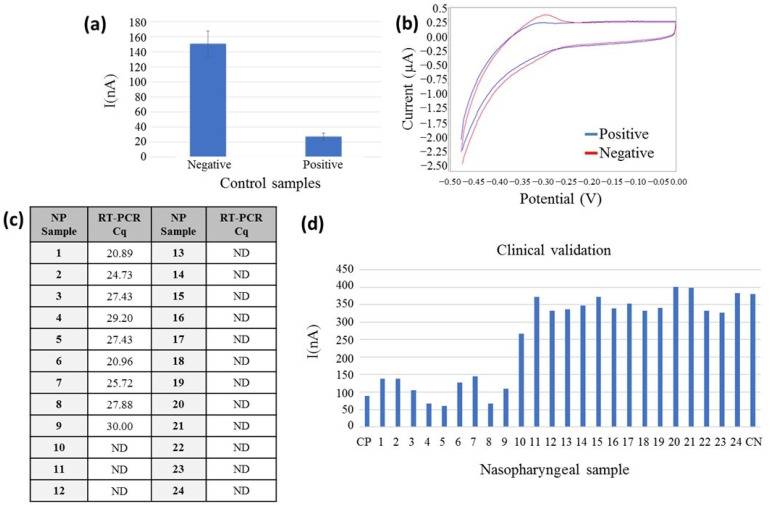
Electrochemical signal detection of the RT-LAMP amplification product as measured on a chip configuration. (**a**) Measures of EC-LAMP amplified products: positive and negative control (n = 10) represented at the bottom and current peak (nA) on the left side, standard deviation represented in bars; (**b**) voltammetry curve of a positive (blue) and negative control (red). Potential is represented in the bottom (V), and the current peak (mA) is represented on the left side; (**c**) Cq values of the correlative NP samples analysed by RT-PCR in the table. ND: not detected. (**d**) On-chip clinical validation results: NP sample numbering represented in the bottom, n = 24; CP: Positive control, CN: Negative control and current peak (nA) on the left side.

The sensor fabrication for on-chip electrochemical measurements was initially tested using positive and negative EC-LAMP products; 1000 copies per reaction of SARS-CoV-2 RNA was used as a positive control, and DNase/RNase-free water was used as a negative control. Following the procedure described in Section 2.2.5, the electrochemical determinations were performed in less than 30 s, obtaining a very reproducible voltammogram, with clear differences between both samples, around five-fold signal difference as the test was validated with five positive and negative samples and with clear characteristic redox peaks of the MB (Figure 5a,b).

For the validation of the electrochemical on-chip configuration, 24 clinical NP samples were measured on-chip. All the NP samples were monitored with the reference method; the Cq of each sample can be observed in the table of Figure 5c. NP samples were heated at 95 °C for 10 min and amplified by the EC-LAMP method, following the detection on the chip. As can be observed in Figure 5d, the electrochemical current signal is lower for the nine positive RT-PCR NP samples. The 15 NP negative samples obtained a signal two-fold higher than the positive ones, demonstrating the feasibility of the developed sensor for the detection of the SARS-CoV-2 virus in heated clinical NP samples. A two-sample t-test was used for comparison of electrical current figures associated with positive (n = 9) and negative (n = 15) samples. It showed a statistical t of 17.08 and a critical value, t_c_, of 2.07 for a 95% confidence level. The electrical current intensity measured shows a statistically significant difference between these two datasets (*p* < 0.001). Differences in absolute values of current intensity between clinical samples in a closed chamber with those studied before obeying both different samples and electroactive volumes in a microfluidic chamber or in an off-chip configuration.

For the last couple of years, where the SARS-CoV-2 pandemic has been the main topic of research, several POC tests have been proposed for the detection of the virus. To compare those tests with the developed EC-LAMP sensor in this work, the characteristics of some POC tests have been summarised in Table 4.

## 4. Conclusions

In conclusion, we have developed a novel electrochemical sensor based on LAMP for the detection of SARS-CoV-2 virus in simple pre-treated NP samples. The extraction process of the sample was successfully avoided by replacing it with an easy-to-process heat treatment. The developed EC-LAMP demonstrated an analytical limit of detection of 62 copies and was 100% specific for SARS-CoV-2 detection. Moreover, the sensor was validated with NP samples with a sensibility and specificity of 100% compared to the reference RT-PCR method. The results obtained confirm that this is a suitable POC sensor for the detection of the SARS-CoV-2 virus in NP samples.

## Figures and Tables

**Table 1:** Inclusive and exclusive viruses for the specificity assay.

Inclusive and Exclusive Virus	Name
Inclusive: *SARS-CoV-2*	*SARS-CoV-2*
Exclusive: Other common respiratory viruses	*Coronavirus OC43*
	*Enterovirus 68*
	*Rhinovirus*
	*MERS coronavirus*
	*Coronavirus SARS-2003*
	*Pool of respiratory virus:* - *Adenovirus 4* - *Coronavirus* - *Influenza A H3N2* - *Influenza B* - *Novel Influenza A H1N1* - *Parainfluenza 1* - *Parainfluenza 2* - *Parainfluenza 3* - *Respiratory syncytial virus (subtype A)* - *Respiratory syncytial virus (subtype B)*

**Table 2:** Results of the limit of detection of the best five sets of primers.

Viral Copies	ND1-1	ND3B	S.45	E.105	ORF1ab.99
1000	10/10 (100%)	10/10 (100%)	10/10 (100%)	10/10 (100%)	10/10 (100%)
500	10/10 (100%)	10/10 (100%)	10/10 (100%)	10/10 (100%)	10/10 (100%)
250	10/10 (100%)	10/10 (100%)	10/10 (100%)	10/10 (100%)	10/10 (100%)
125	10/10 (100%)	10/10 (100%)	9/10 (90%)	9/10 (90%)	9/10 (90%)
62	9/10 (90%)	10/10 (100%)	10/10 (100%)	5/10 (50%)	5/10 (50%)
41	8/10 (80%)	8/10 (80%)	6/10 (60%)	NT ^1^	NT
31	6/10 (60%)	8/10 (80%)	6/10 (60%)	3/10 (30%)	2/10 (20%)
16	6/10 (60%)	5/10 (50%)	4/10 (40%)	NT	NT

^1^ Not tested.

**Table 3:** Comparison of PCR-positive NP sample pre-treatments.

	Column-Based	95 °C 10 min	Extraction-Free
RT-LAMP	100%	100%	73%
Mean TTD	11.64	11.62	18.60
Sample pre-treatment time	40 min	10 min	0 min

**Table 4:** Comparison of different POC tests for the detection of SARS-CoV-2 in NP samples.

Method	Extraction-Free	Detection Method	LoD	Disadvantage	Reference
LAMP-RPA	Yes	Colourimetric	5 copies	Two temperatures	[33]
LAMP-CRISPR-Cas-Lateral-flow	No	Colourimetric	20 copies	Extraction step Two temperatures	[34]
CRISPR-Cas	No	Electrochemical	50 fM	Extraction step Less sensitive	[35]
LAMP	No	Diffusometry analysis	20 pg	Extraction step Works with cDNA	[36]
Lateral-flow immunoassay	Yes	RAMAN	0.03 ng/mL	Less sensitive	[37]
LAMP	Yes	Electrochemical	62 copies	-	This work

## Data Availability

The data presented in this study are available on request from the corresponding author.
